# AlphaFold2 and CryoEM: Revisiting CryoEM modeling in near-atomic resolution density maps

**DOI:** 10.1016/j.isci.2022.104496

**Published:** 2022-05-30

**Authors:** Corey F. Hryc, Matthew L. Baker

**Affiliations:** 1 Department of Biochemistry and Molecular Biology, Structural Biology Imaging Center, McGovern Medical School at The University of Texas Health Science Center at Houston, 6431 Fannin Street, Houston, TX 77030, USA

**Keywords:** Computational molecular modelling, Biochemistry, Structural biology

## Abstract

With the advent of new artificial intelligence and machine learning algorithms, predictive modeling can, in some cases, produce structures on par with experimental methods. The combination of predictive modeling and experimental structure determination by electron cryomicroscopy (cryoEM) offers a tantalizing approach for producing robust atomic models of macromolecular assemblies. Here, we apply AlphaFold2 to a set of community standard data sets and compare the results with the corresponding reference maps and models. Moreover, we present three unique case studies from previously determined cryoEM density maps of viruses. Our results show that AlphaFold2 can not only produce reasonably accurate models for analysis and additional hypotheses testing, but can also potentially yield incorrect structures if not properly validated with experimental data. Whereas we outline numerous shortcomings and potential pitfalls of predictive modeling, the obvious synergy between predictive modeling and cryoEM will undoubtedly result in new computational modeling tools.

## Highlights

• AlphaFold2 can be combined with cryoEM density maps for macromolecular modeling
• Even at lower resolutions, AlphaFold2 and cryoEM can generate testable models
• Predictive modeling must be validated by experimental data for reliability

## Graphical abstract

**Figure:**
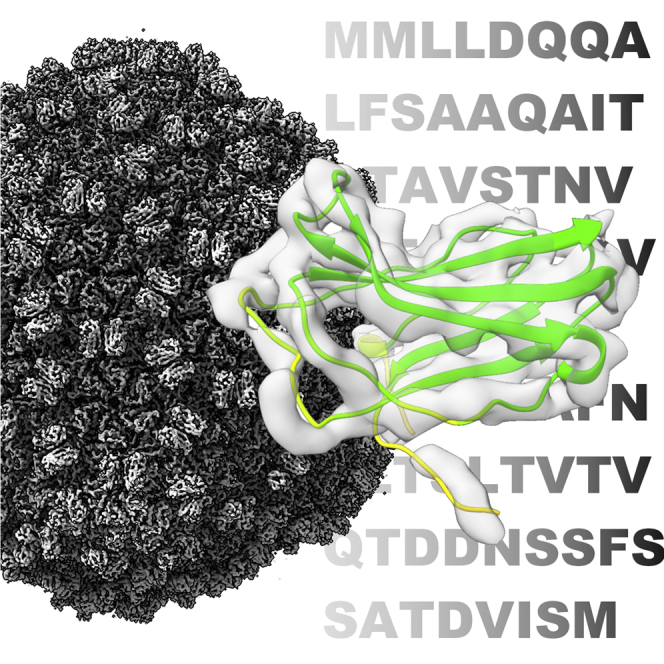

## Introduction

Over the past decade, single-particle electron cryomicroscopy (cryoEM) has become an increasingly popular and powerful tool in understanding the structure and function of macromolecular assemblies (Callaway, 2020; Cressey and Callaway, 2017; Wigge et al., 2020). Whereas density maps at near-atomic resolutions were relatively uncommon a decade ago, it is now quite common (Yao et al., 2020) to achieve 3D reconstructions at better than a 4-Å resolution, where robust and reliable models can be built directly from the density map (Henderson et al., 2012; Lawson et al., 2021). State-of-the-art reconstructions are even pushing toward a 1-Å resolution, resolving densities for individual atoms, ions and waters (Nakane et al., 2020; Xie et al., 2020; Yip et al., 2020; Zhang et al., 2020). The appropriately named “resolution revolution” was driven in large part by technological advancements in electron microscopes, imaging hardware and data processing tools (Egelman, 2016). Today, cryoEM plays a pivotal role in medicine and human health, providing structure-function detail for many large macromolecular assemblies, including ribosomes (Kišonaitė et al., 2022), ion channels (Liao et al., 2013), G-protein coupled receptors (Liang et al., 2017), CRISPR (Lapinaite et al., 2020), and SARS-CoV-2 (Yao et al., 2020).

Nearly all current *“de novo”* cryoEM modeling tools are based on a common concept; visible features in the density map are used as anchors to establish the protein fold, followed by the optimization and refinement of atom positions using a variety of force fields and chemical restraints (Hryc and Baker, 2021). At high resolutions (<2.5 Å), these processes are relatively robust and can build reliable models, whereas at lower resolutions, the “standard” approaches are often less accurate and require manual optimization. Regardless, modern cryoEM modeling tools provide an excellent platform for interpreting experimental data at the atomic level.

Like the revolution in cryoEM, predictive modeling is undergoing a similar “revolution” ushered in by softwares such as AlphaFold (AlphaFold2) (Jumper et al., 2021) and RoseTTAFold (Baek et al., 2021) that are based on artificial intelligence and machine learning methods. Molecular models generated from the amino acid sequence alone have shown tremendous accuracy in CASP14 (https://predictioncenter.org/casp14) and have dramatically increased the value of predictive models in addressing the structure and function of individual proteins. It has already been shown that AlphaFold2 and RoseTTAFold models can be used for molecular replacement in X-ray crystallography (Millán et al., 2021) or combined with cryoEM density maps (https://doi.org/10.1101/2022.01.07.475350).

The use of computational and predictive modeling for building atomic models in cryoEM density maps is not necessarily a new concept (Baker et al., 2006; Topf et al., 2005), though, before the incorporation of artificial intelligence and machine learning, predictive modeling has had relatively low accuracy and reliability for proteins in macromolecular complexes. The success of AlphaFold2 and RoseTTAFold has undoubtedly inspired a fresh approach for model building in cryoEM density maps; however, the utility, limits, and expectations of such a combined approach must be first evaluated. In this work, we examine the application of predictive modeling with respect to a variety of data sets from past cryoEM modeling challenges. Specifically, we explore how accurate and effective predictive models may be in interpreting cryoEM density maps, as well as begin to establish boundaries when combining these two methods. Furthermore, this work provides a retrospective analysis of earlier cryoEM modeling efforts, identifying possible successes and failures, as well as highlighting areas of future research in cryoEM modeling.

## Results

In order to assess the utility of current predictive modeling methods for constructing atomic models in cryoEM density maps, we examined a set of community established standard density maps taken from the past three cryoEM modeling challenges: the 2021 Ligand Model Challenge, the 2019 Model Metrics Challenge, and the 2016 Model Challenge (https://challenges.emdataresource.org/). In total, these three challenges provided 12 unique biological data sets ranging from 1.8 to 4.5 Å in resolution and represent a variety of macromolecular complexes, making them ideal candidates to evaluate the accuracy of predictive modeling in the context of cryoEM density maps. AlphaFold2 was run on the sequences for each protein subunit in the individual challenge data sets, with the exception of EMDB: EMD-2847, a 70-s ribosome structure with over 50 individual protein subunits. For EMDB: EMD-2847, six random chains were selected for modeling with AlphaFold2. All AlphaFold2 models were assessed on multiple criteria, including predictive modeling confidence, overall model quality, fit to density and accuracy when compared with the reference structure.

**Figure 1:**
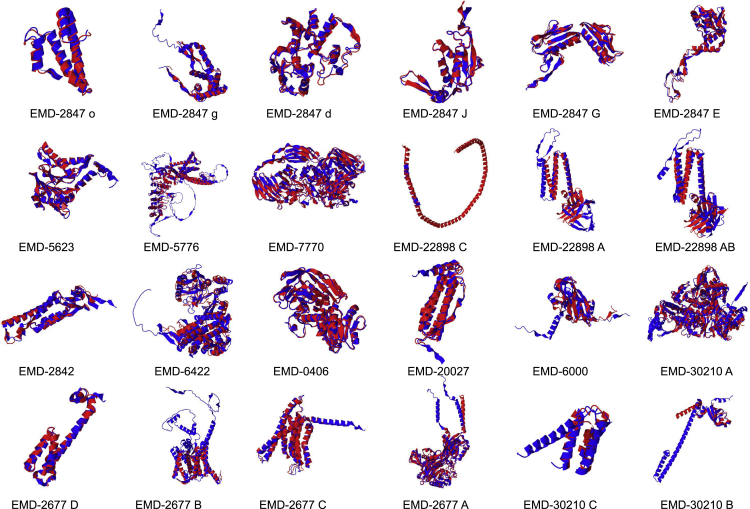
AlphaFold2 and the cryoEM challenge data sets A selected set of AlphaFold2 models (blue) are shown superimposed on the experimentally derived reference structures (red). The structural alignments are derived from the TM-score alignments.

The primary metric of AlphaFold2 is the pLDDT (predicted Local Distance Difference Test) score, which gives a measure of predictive model quality (Mariani et al., 2013). Generally, models with pLDDT scores above 70 are considered “confident” and scores below 50 are considered “very low confidence”; scores between 50 and 70 are borderline low confidence but may be correct or have parts that are correct. Of the 23 chains, only three had pLDDT scores below 70 and only one of those was below 50, indicating that AlphaFold2 was mostly able to predict these structures with high confidence (Figure 1 and Table 1).

**Table 1:** AlphaFold2 predictive modeling on the cryoEM challenge data sets

EMDB/PDB ID (resolution)	Length (aa)	Molprobity score	Clash score	Ramachandran outliers (%)	Rotamer outliers (%)	Map/model CC	pLDDT	TM-score	RMSD (Å)
EMD 0406/6NBB (2.9 Å)	375	1.88	15.03	0.54	0.97	0.7825	96.77	0.7754	3.790 (374)
EMD 2677/5A63 (4.5 Å)									
A	709	2.22	19.81	2.40	0.65	0.8013	90.96	0.9795	1.581 (665)
B	467	3.56	52.33	16.99	5.88	0.5760	66.51	0.9472	1.420 (215)
C	265	1.87	17.59	1.14	0.93	0.7714	93.06	0.9753	0.985 (243)
D	101	1.94	25.78	0.00	1.12	0.8004	93.12	0.9417	1.059 (100)
EMD 2842/4UDV (3.3 Å)	159	2.47	15.83	3.82	2.16	0.6328	88.55	0.5901	3.935 (153)
EMD 2847/5AFI (2.9 Å)	201	1.77	18.94	0.50	0.0	0.5792	93.41	0.9420	1.364 (201)
E	177	1.80	20.28	0.0	0.72	0.7169	91.34	0.6339	3.801 (176)
G	142	1.91	24.46	0.0	0.86	0.7076	93.62	0.9687	0.945 (142)
J d<	206	1.71	16.03	0.0	0.0	0.6944	92.32	0.6757	3.752 (205)
g	179	2.68	16.06	6.78	3.40	0.6235	92.16	0.6108	3.852 (151)
o	89	1.48	8.87	0.0	0.0	0.687	92.80	0.4674	3.818 (88)
EMD 5623/3J9I (3.3 Å)	233	1.62	12.83	0.43	0.0	0.8474	94.32	0.9694	1.076 (224)
EMD 5778/3J5P (3.275 Å)	838	3.20	30.04	14.71	3.91	0.6057	69.99	0.9545	2.963 (592)
EMD 6000/3J7N (3.8 Å)	189	3.39	60.10	11.76	3.42	0.6241	66.90	0.7805	3.649 (149)
EMD 6422/3CAU (4.1 Å)	548	2.06	15.28	2.01	1.20	0.6460	92.21	0.7346	5.354 (526)
EMD 7770/6CVM (1.9 Å)	1,021	1.97	22.77	0.29	0.34	0.7100	94.45	0.8837	3.850 (1020)
EMD 20027/3AJO (2.32 Å)	183	1.86	13.73	2.21	0.61	0.7675	95.10	0.6305	3.785 (172)
EMD 22898/3KJR (2.08 Å)									
A	284	3.62	30.09	18.79	12.84	0.2329	48.16	0.4580	11.507 (193)
AB∗	568	3.58	27.67	15.96	13.04	0.2756	39.11	0.6035	9.469 (193)
C	210	2.57	5.53	4.81	15.68	0.2674	72.43	0.1247	2.756 (31)
EMD 30210/7KVB (2.5 Å)									
A	951	2.65	35.57	3.48	1.55	0.5375	88.85	0.8402	4.675 (834)
B	207	2.26	17.64	0.49	1.70	0.4233	85.04	0.4654	5.535 (114)
C	92	2.90	33.45	3.33	3.49	0.5366	80.14	0.3089	3.899 (63)

AlphaFold2 was run on each protein subunit from the individual cryoEM challenge data sets, with the exception of EMDB: EMD-2847. For EMDB: EMD-2847, a 70s ribosome structure with over 50 individual protein subunits, six random chains were selected for modeling with AlphaFold2. The resulting models were assessed for model quality including the Molprobity score, clash score, Ramachandran outliers, rotamer outliers), fit to density (map/model cross correlation), predicted level of confidence (pLDDT), and similarity to the reference model (TM-score, RMSD). In the final column, the number listed in parenthesis after RMSD refers to the total number of matched residues between the model and reference structures. The resolution for each map is listed in the first column after the EMDB and PDB IDs. For EMDB: EMD-22898, the asterisk indicates AlphaFold2 was run on a homodimer of chain A, denoted as AB in the table and text.

The model quality for all the predictive models was assessed with Molprobity (Williams et al., 2018). As expected, AlphaFold2 produced reasonably good stereochemical models, though some models did have relatively large clash scores and Ramachandran outliers. Regardless, these models would be considered of sufficient quality for further refinement into the corresponding near atomic resolution density map.

### Modeling challenge data

As reference models were available for all of the challenge data sets, the model accuracy was directly assessed by comparing the AlphaFold2 models with the experimentally derived models. Evaluation of the models used the TM-score (Template Modeling Score) that assessed fold similarity (values 0–1, where 1 indicates a perfect match) (Zhang and Skolnick, 2004), and Cα RMSD with the reference model, further revealing that AlphaFold2 was able to accurately predict the structure of the individual components based purely on sequence. Moreover, fitting of the models to the density map also revealed that AlphaFold2 was indeed able to nearly perfectly capture the structure of the individual protein components, with the notable exception of one data set.

The exception to AlphaFold2’s success was EMDB: EMD-22898, the SARS-CoV-2 ORF3a putative ion channel in nanodisc at a 2.1-Å resolution. For chain A (284aa), the AlphaFold2 model had an RMSD of 11.507 Å and a TM-score of 0.4580 when compared with the reference structure (5AFI), indicating major differences at the fold level between the predicted model and reference structure. Chain A from EMDB: EMD-22898 also had a pLDDT score of 48.16, indicating an overall low confidence in the model. Upon visualization of the model and reference structure, the major source of model variation was due to the orientation of the helical domain relative to the β-sheet domain (Figure 1). Owing to the orientation of the two domains for chain A in EMDB: EMD-22898 being different in the predictive model than the reference structure, the rigid-body fitting of the model did not produce a “good” fit to the density.

The published model for EMDB: EMD-22898 is homodimeric, with two identical copies of the protein in question forming the putative ion channel. In an attempt to determine if the dimeric state of the protein was required to achieve the proper orientation of the two domains, AlphaFold2_advanced was run using two copies of chain A (labeled as AB in Table 1). The resulting homodimeric AlphaFold2 model was slightly improved when compared with the reference structure, reducing RMSD to 9.469 Å and improving the TM-score to 0.6035. Notably, the pLDDT score dropped to 39.11 even though the overall predictive model agreed better with the reference structure. However, the homodimeric complex also did not have correct orientations of the domains, making the overall fit to density only slightly better than the fitting of the monomeric chain A.

Unlike chain A, chain C of EMDB: EMD-22898 had a relatively high pLDDT score, a good Molprobity score and a low RMSD when compared with the reference structure; however, the TM-score and fit to density would indicate a poor model. This aberration in the scores was based on the density of chain C only accommodating 30–35 residues of the predictive model, though the full 210 amino acid sequence was modeled with AlphaFold2.

In addition to EMDB: EMD-22898, EMDB: EMD-30210, the SARS-CoV-2 RNA-dependent RNA polymerase, had relatively low fit to density scores (below 0.5), though the pLDDT, TM-score, and RMSD scores indicated that AlphaFold2 was successfully at modeling the three chains in the map. Upon further examination, whereas there are some differences between the model and the reference structures, the majority of the poor fit comes from AlphaFold2 modeling regions that were not resolved in the density map. A similar drop in fit to density was observed in EMDB: EMD-5778, the structure of TrpV1, where over 200 amino acids were modeled by AlphaFold2, but were not visible in the density map.

Outside of EMDB: EMD-22898 and EMDB: EMD-30210, which were published in 2020, all of the challenge data sets would likely have been well represented in the AlphaFold2 training set. In an effort to gain an understanding of how AlphaFold2 might perform on other cryoEM data sets, three previously reported virus data sets, reconstructed between 3- and 5-Å resolution were selected for testing. Key to selection of these data sets were the following: 1) none of the data sets had been previously modeled with AlphaFold2; 2) models for these proteins were not included in the AlphaFold2 training/template library; 3) the data sets contained at least components with a related, but non-identical structure in the PDB; 4) the data sets contained multiple proteins; and 5) the complete data sets were available.

### Bacteriophage ε15

Tailed double-stranded DNA (dsDNA) bacteriophages were present billions of years before cellular life diverged, and are one of the most abundant life forms on the planet today. NMR, X-ray crystallography, and single-particle cryoEM have been used extensively to determine the structures of bacteriophages. In 2008, the first near-atomic resolution cryoEM structure of ε15, an infectious bacteriophage, was reported (Jiang et al., 2008). Using an *ad hoc* implementation of feature registration and sequence matching, a trace of the Cα backbone of gp7, the 335 amino acid (aa) major capsid protein, and gp10 (111 aa), a second capsid protein decorating the outer surface of the capsid shell, were determined. Despite low sequence similarity, gp7 shared considerable structural homology to the capsid protein from bacteriophage HK97 and other major capsid proteins from tailed dsDNA bacteriophages (Morais et al., 2005). Whereas capsid decoration proteins had previously been reported in other bacteriophages, no related sequences or structures could be identified for gp10. Several years after the initial Cα backbone models, a higher resolution cryoEM map was obtained that allowed for the construction of a refined, full-atom model for the entire viral capsid (PDB: 3J40, EMDB: EMD-5678) (Baker et al., 2013).

### ε15 major capsid protein

**Figure 2:**
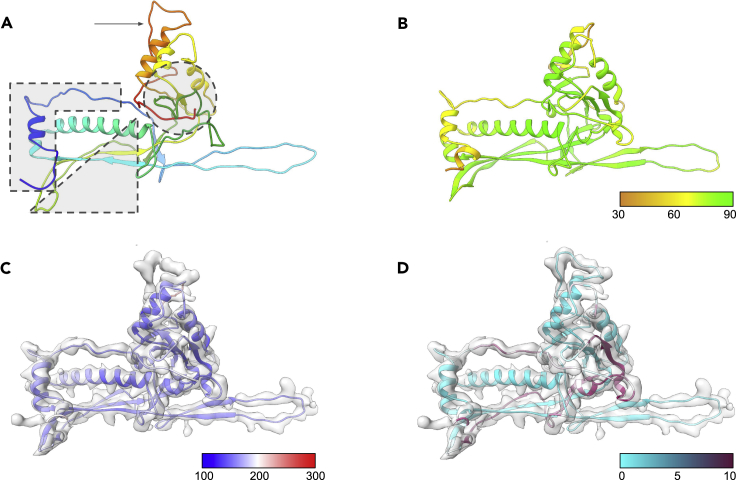
Modeling of Epsilon15 gp7 (A) The structure of a single gp7 subunit (PDB: 3J40_B) is shown. The model is colored from the N-terminus (blue) to the C-terminus (red). The N-terminus is enclosed by a dashed box, the long β-sheet is enclosed by a dashed triangle, the internal β-sheet is shown enclosed by a dashed circle, and the large hexonal loop is marked with an arrow. (B) The AlphaFold2 model for gp7 is shown as a ribbon colored by the pLDDT value. (C) The AlphaFold2 model for gp7 after real-space refinement in Phenix is shown superimposed on the density map for a single gp7 subunit and colored based on B-factor. (D) The final model for gp7 based on the AlphaFold2 prediction and refined against the density with Phenix and Coot is shown. The model is colored by RMSD, comparing it with the published structure, 3J40_B.

ε15 gp7, the 335aa major capsid protein, was first modeled with AlphaFold2 and refined against the density map. The initial AlphaFold2 model for gp7 was very similar to the published structure (PDB: 3J40, chain B), as well as the aforementioned HK97 capsid protein structure (Figures 2A and 2B, Table 2). Statistics for the AlphaFold2 gp7 model were as good or better than the published structure and the mean pLDDT score was 77.80, indicating a relatively well-predicted model. Only the N-terminal ∼40 residues and residues 298–312 had pLDDT scores below 70. As discussed below, these residues had a large RMSD when compared with the published structure and had a relatively poor fit to the density. However, the overall results for gp7 were very much on par with the results obtained from the challenge data sets.

**Table 2:** ε15 gp7 model statistics

	3J40_B	AlphaFold2 model	AlphaFold2 model with RSR	AlphaFold2 model with RSR + Coot
Composition				
Atoms	2,588	2,587	2,587	2,587
Residues	335	335	335	335
Molprobity score	3.96	2.69	1.70	1.77
Clash score	27.68	27.71	8.58	9.55
Ramachandran plot				
Outliers	12.31	3.00	0.60	0.30
Allowed	25.23	5.41	3.00	3.60
Favored	62.46	91.59	96.40	96.10
Rotamer outliers	30.66	2.19	0.00	0.00
Cbeta deviations	1.30	0.00	0.00	0.00
Bonds				
Length	0.006	0.017	0.004	0.004
Angles	1.504	1.884	0.733	0.663
B-factor (mean)	67.45	–	156.05	145.70
pLDDT (mean)	–	77.80	–	–
Map–model CC	0.64	0.54	0.77	0.79
FSC (0.143)	3.8	4.0	3.2	3.2
TM-score	–	0.7491	0.7710	0.7944
RMSD (paired residues)	0	5.984 (335)	5.628 (335)	4.872 (335)

In this and rest of the tables, root-mean-square deviation (RSMD) is computed between pairwise matching residues. Unless otherwise indicated, RMSD is computed across all residues.

The initial comparison of the AlphaFold2 model with 3J40_B yielded an RMSD of 5.984 Å. A single round of real-space refinement with morphing and simulated annealing improved the model agreement slightly, reducing the RMSD to 5.628 Å (Figure 2C). In an attempt to further refine the model, manual optimization in Coot and a subsequent round of real-space refinement reduced the overall RMSD to 4.872 Å while also improving the fit to density (Figure 2D). A complete summary of model statistics and map/model agreement can be found in Table 2.

Despite the relatively high RMSDs, the AlphaFold2 model closely resembled the published structure of gp7 except in four regions. In the published structure, the N-terminal ∼40 residues consist of a short helix followed by an elongated loop that runs parallel to the central, long helix (box in Figure 2A). In the AlphaFold2 model, a second short helix is introduced at the N-terminus, preceding the observed helix in 3J40_B, resulting in a 1-3 amino acid shift in residue assignment in the elongated loop. After real-space refinement, RMSD in the N-terminal helical region decreases, though the register shift is still present in the elongated loop. Despite an average RMSD of over 5 Å, the overall fit to density of the elongated loop is improved over the published model and the initial AlphaFold2 model.

The second major difference occurs in the outer portions of the primarily β-sheet region immediately following the long, central helix (circle in Figure 2A). This region consists of residues 134–171, 244–258, and 324–335. Two of these strands, residues 134–171 and 324–335, are nearly identical to the overall fold of the published structure though the sequence is again shifted. In residues 134–171, there is a 1-4 amino acid registration difference; residues 165–168 in the AlphaFold2 model are particularly distorted, but bring the sequence back into register by residue 170. Whereas the AlphaFold2 model for residues 165–168 does not appear to fit the density as well as the published model, they consist of Ala, Gly, Gly, Ser on the outermost surface, and, in fact, may be flexible and have poorly resolved density in the map. Similarly, residues 324–326 are responsible for a register shift in the C-terminal loop, though it improves considerably after real-space refinement. Residues 244–258 of 3J40_B contains a short helix (residues 244–250) followed by a loop, whereas the AlphaFold2 model essentially contains one single loop. The density map appears to indicate the presence of a helix-like feature corresponding to residues 244–248; the density immediately after this is less clear. After real-space refinement, the AlphaFold2 model maintains the overall loop structure but moves closer to the published structure. Like the other regions, the agreement with the density is improved over the initial AlphaFold2 model, as well as the previously published structure.

Running parallel to the central helix, three anti-parallel strands form a long, prominent β-sheet (triangle in Figure 2A). The “bottom” strand, formed by two smaller strands separated by a short loop, ends in a loop near the N-terminus. This bottom strand (residues 182–215) also has a register shift of up to four residues when compared with the published model. The initial shift in registration occurs at residues 182–185 that form a short loop before the first strand. Registration with the published model is again established between residues 208 and 214 that form an extended loop between the bottom and middle strand of the β-sheet. The AlphaFold2 model for this region significantly improves the hydrogen bonding in the β-sheet; however, the density is not well resolved and, after real-space refinement, some of the hydrogen bonding in the bottom strand is lost.

Perhaps the largest area of difference occurs from residues 298 to 312 (arrow in Figure 2A). The AlphaFold2 model depicts these residues with two small anti-parallel strands forming a β-sheet. The published model contains an extended loop for this region and corresponding residues in the two structures are up to ∼17 Å apart. When compared with the density, the AlphaFold2 model for this region does not fit, nor does the density appear to be consistent with β-strand features. Simple real-space refinement improves the fit to density for the original AlphaFold2 model slightly, but still largely maintains the overall architecture of the AlphaFold2 prediction. Unlike the aforementioned issues, manual refinement of this region was needed to bring the model into agreement with the density map. After refinement in Coot, the average RMSD between the AlphaFold2 model and the published structure in this region drops below 1 Å. It is interesting to note that this region is located proximal to the local six-fold axes, where the same loop from the six hexonal subunits may interact and this interaction is not captured by AlphaFold2.

### ε15 accessory protein

**Figure 3:**
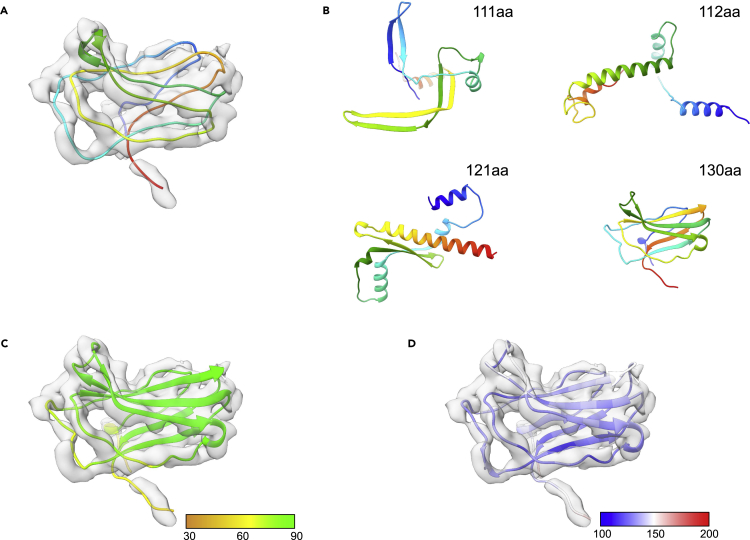
Modeling of Epsilon15 gp10 (A) The structure of a single gp10 subunit (PDB: 3J40_H) is shown superimposed on corresponding density. The model is colored from the N-terminus (blue) to the C-terminus (red). (B) AlphaFold2 models for four potential candidate accessory proteins gp10 sequences, all with varying sequences, are shown as ribbons and colored from the N-terminus (blue) to the C-terminus (red). (C) The AlphaFold2 model for the 130aa protein superimposed on the gp10 density is shown colored by pLDDT. (D) The real-space refined 130aa gp10 model is shown superimposed on the corresponding density and is colored based on B-factor.

In the original ε15 paper (Jiang et al., 2008), an “accessory” capsid protein was discovered after modeling gp7; the gp7 structure left a sizable unmodeled density, presumed to be a yet-to-be-identified structural protein, on the surface of the capsid. A proteomic analysis of infectious ε15 viral particles putatively identified gp10, a 111 amino acid (∼12 KDa) structural protein, a previously unknown structural protein. At the time of the publication, no additional ∼12kDa structural proteins were known, leaving gp10 as the only candidate for the unmodeled density. Secondary structure prediction of the gp10 sequence largely supported the observed β-strands based in this region of the density map, though it was not until a subsequent higher resolution ε15 density map was obtained that a model for this density could be constructed based on the gp10 sequence (Figure 3A) (Baker et al., 2013).

In continued evaluation of AlphaFold2 with ε15, a model for the 111 amino acid gp10 sequence was generated and appeared, once again, to have predictive and model quality metrics on par with what was observed in the challenge data. However, the AlphaFold2 model bore little resemblance to the published structure or the density map, outside of two long strands (Figure 3B, top left panel). Despite good model quality and high levels of confidence, the failure of AlphaFold2 to produce a model consistent with the experimental density map and gp10 model meant either 1) AlphaFold2 generated a “good” but inaccurate model or 2) the original assignment of the accessory protein to the 111aa gp10 sequence was wrong.

Since the original ε15 models, it was discovered that gp10 was not the only ∼12KDa protein in the ε15 genome. In fact, three additional unique proteins were identified with similar molecular weights to gp10: a 112, 121, and 130 amino acids protein (Figure S1). These sequences represented unique proteins and no structure for any of these new sequences was known. To determine if one of these sequences could account for the gp10 density (and suggest that the original assignment of gp10 to the second capsid protein was wrong), these three proteins were submitted to AlphaFold2 for modeling (Figure 3B). Like the 111 amino acid protein, the 112 and 121 amino acid proteins had no similarity to the published structure or the density map. Both the 112 and 121 amino acid proteins were predicted to contain large prominent helices that would have been clearly observed in the density map. Despite being 19 residues longer and having almost no sequence similarity to the 111 amino acid sequence, the AlphaFold2 model for the 130 amino acid sequence was consistent with the density map, clearly matching the six prominent β-strands in the map and appeared to have the correct overall fold (Figures 3C and 3D).

The AlphaFold2 model for the 130 amino acid sequence was not as “good” as the other three models in terms of measurable model quality and predicted accuracy, though all four models had average pLDDT scores above 80 (Table 3, Figure 3C). Visual inspection and fit to density (map–model cross correlation) were easily the biggest discriminators of the “correct” model from four candidate structures. For the 130 amino acid model, the overall quality and fit to density were improved with real-space refinement, surpassing the published model in almost all categories (Figure 3D). The results here would seem to indicate that the 111 amino acid sequence was indeed incorrectly assigned to the second capsid protein and that the 130 amino acid sequence likely corresponds to the second capsid protein density.

**Table 3:** ε15 gp10 model statistics

	3J40_H	111aa	112aa	121aa	130aa	130aa refined
Composition						
Atoms	850	850	870	929	952	952
Residues	111	111	112	121	130	130
Molprobity score	4.22	2.00	1.73	1.75	2.61	2.27
Clash score	47.31	14.97	12.01	10.29	22.16	18.56
Ramachandran plot						
Outliers	13.76	3.67	0.00	1.68	4.69	0.0
Allowed	34.86	0.92	2.73	1.68	6.25	8.59
Favored	51.38	95.41	97.27	96.64	89.06	91.41
Rotamer outliers	28.42	0.00	0.00	0.00	1.82	0.00
Cbeta deviations	1.96	0.00	0.00	0.00	0.00	0.00
Bonds						
Length	0.006	0.019	0.015	0.019	0.016	0.004
Angles	1.760	2.202	1.420	1.941	1.933	0.772
B-factor (mean)	106.00	–	–	–	–	135.79
pLDDT (mean)	–	80.29	93.68	95.18	83.22	–
Map–model CC	0.71	0.10	0.16	0.19	0.61	0.81
FSC (0.143)	4.0	2.7	2.6	7.1	4.1	3.3
TM-score	–	0.1509	0.1337	0.1352	0.1914	0.1912
RMSD (residues)	0	21.425 (111)	9.404 (30)	0.254 (3)	14.819 (48)	19.290 (98)

### Bacteriophage Syn5

The most abundant cyanobacteria in the oceans, Synechococcus and Prochlorococcus fix ∼30% of CO_2_ in the atmosphere through photosynthesis. Similarly, cyanophages, bacteriophages infecting cyanobacteria, are extraordinarily abundant and crucial components of the marine ecosystem. Cyanophage Syn5, which infects Synechococcus, is a dsDNA bacteriophage belonging to the Podoviridae family and has a T7 bacteriophage-like genome organization. In 2014, the ∼4.7-Å resolution structure of Syn5 from single-particle cryoEM was reported (PDB: 4BMI, EMDB: EMDB-5954) (Gipson et al., 2014). A model for the major capsid protein, gp39, revealed structure similarity to the major capsid proteins of other tailed dsDNA bacteriophages (like HK97, ε15, etc.), despite only minimal sequence similarity. As with ε15, additional capsid proteins were detected and tentatively assigned as gp55 and gp58 to the density map based on size and secondary structure content. However, models for gp55 and gp58 were not generated owing to the limited resolution and the lack of suitable structural homologues.

### Syn5 gp39

**Figure 4:**
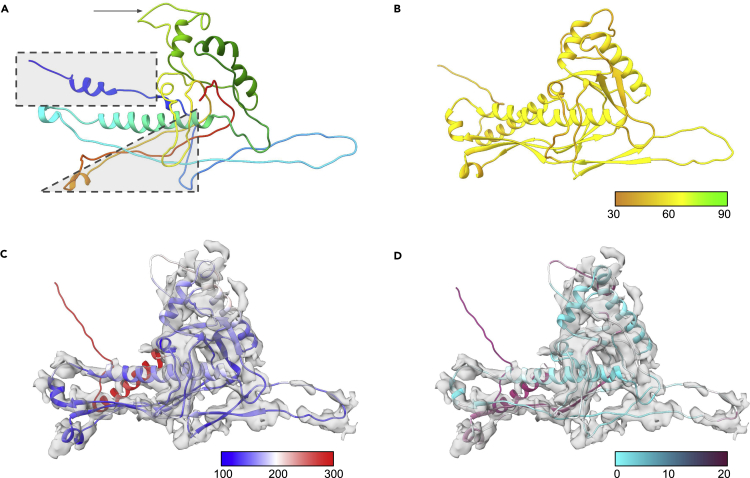
Modeling of Syn5 gp39 (A) The structure of a single gp39 subunit (PDB: 4BMI_A) is shown colored from the N-terminus (blue) to the C-terminus (red). The N-terminus is enclosed by a dashed box, the long β-sheet is enclosed by a dashed triangle, and the large hexonal loop is marked with an arrow. (B) The AlphaFold2 model for gp39 is shown colored by pLDDT. (C) The AlphaFold2 model for gp39 after real-space refinement in Phenix is shown superimposed on the density map for a single subunit and colored based on B-factor. (D) The final model for gp39 based on the AlphaFold2 prediction and refined against the density with Phenix and Coot. The model is colored by RMSD, comparing it with the published structure 4BML_A.

At the reported resolution, only a Cα backbone model could be constructed for gp39. For the purpose of this work, the Cα backbone model was converted into a full-atom model and refined with one round of real-space refinement (using morphing and simulated annealing options) in Phenix (Figure 4A). Prediction of the gp39 (332aa) structure with AlphaFold2 produced models similar to the experimentally derived model and consistent with the aforementioned HK-97-like fold (Figure 4B and Table 4). Three of the five models had average pLDDT scores above 60, with most of the core structure elements having pLDDT scores between 70 and 75.

**Table 4:** Syn 5 model statistics

	4BML_A	gp39	gp39 refined	gp55	gp58	gp58 refined
Composition						
Atoms	2,298	2,469	2,469	1,170	1,162	1,162
Residues	309	332	332	156	169	169
Molprobity score	2.47	3.18	2.30	3.05	3.66	3.50
Clash score	17.57	33.01	19.05	29.22	51.95	37.10
Ramachandran plot						
Outliers	1.63	10.00	0.30	9.74	22.75	4.79
Allowed	17.59	9.39	8.79	9.09	12.57	28.14
Favored	80.78	80.61	90.91	81.17	64.97	67.07
Rotamer outliers	0.00	3.89	0.00	3.17	5.83	5.83
Cbeta deviations	0.00	0.00	0.00	0.00	0.00	3.95
Bonds						0.009
Length	0.003	0.019	0.003	0.017	0.024	
Angles	0.850	3.071	0.738	3.304	5.688	
B-factor (mean)	88.87	–	175.17	–	–	22.26
pLDDT (mean)	–	60.99	–	76.59	46.36	–
Map–model CC	0.45	0.23	0.55	0.04	0.08	0.45
FSC (0.143)	4.4	7.0	4.2	9.4	6.0	4.1
TM-score	–	0.5575	0.5529	–	–	–
RMSD (paired residues)	0	10.326	9.827	N/A	N/A	N/A

Despite the somewhat lower pLDDT and model quality scores in comparison with the challenge data sets, all five of the models visually appeared to fit the density map. The overall RMSD between the experimental structure and the five AlphaFold2 models was ∼10 Å, with the best-fitting model having an RMSD of 10.326 Å. Real-space refinement model greatly improved the FSC and cross-correlation, going from 7 to 4.2 Å and 0.23 to 0.55, respectively (Figure 4C). The overall RSMD also improved by ∼0.5 Å, correcting some small fit to density issues (Figure 4D). However, as was the case for gp7 of ε15, the majority of the differences between the AlphaFold2 and experimental models occurred in the N-terminal domain, hexonal loop, and the large three-stranded β-sheet (Figure 4A, shown by the dashed rectangle, dashed triangle, and arrow, respectively). In the AlphaFold2 model, the N-terminal domain is modeled as a long helix just below the central long helix. This arrangement is similar to the bacteriophage P22 procapsid structure major capsid protein, which also has an HK97-like fold (Chen et al., 2011; Kaelber et al., 2017). In examining the experimental and refined AlphaFold2 models for gp39, there was unmodeled density corresponding to the location of the N-terminal helix of the AlphaFold2 model and the experimentally derived model does have considerable clashes with neighboring subunits at the N-terminus. This suggests that the AlphaFold2 model for gp39, at least in this region, may better reflect the actual fold of gp39 in the density map. As for the hexonal loop, AlphaFold2 produces a loop that is tucked up against the rest of the structure, whereas the experimental model and density suggest that this loop is extended. In the large three stranded β-sheet, a sequence registration error is largely responsible for the majority of the differences, though the small helix that occurs between the two bottom strands of the β-sheet is shifted.

### Syn5 gp55 and gp58

**Figure 5:**
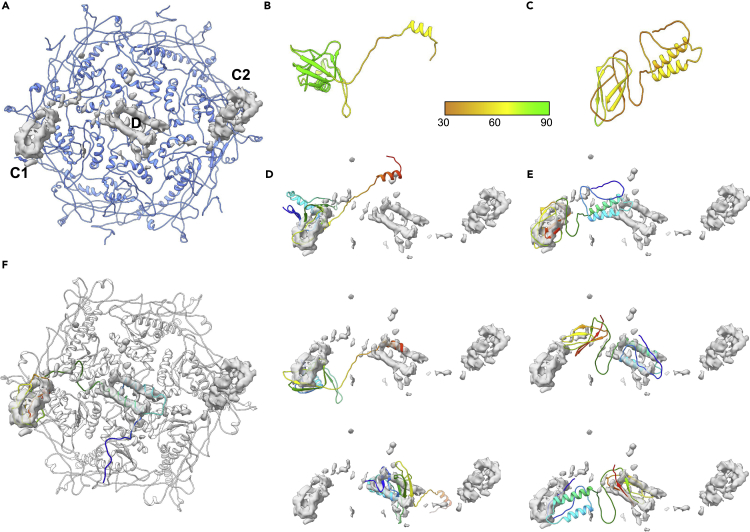
Modeling of Syn5 extra density (A) A model of the gp39 hexon is shown in blue. Subtracting the density belonging to gp39 revealed the unmodeled denisites C1/C2 and D. (B) The AlphaFold2 model for gp55 is shown colored based on pLDDT. (C) The AlphaFold2 model for gp58 is shown colored based on pLDDT. Extra density with C1/C2 and D labels. (D) The AlphaFold2 model for gp55, colored from the N-terminus (blue) to the C-terminus (red), is shown fit to the unmodeled density. In the three panels, the top panel shows the N-terminal domain fit to the C1/C2 density, the middle panel shows the C-terminal domain fit to the D density, and the lower panel shows the N-terminal domain fit to the D density. (E) The AlphaFold2 model for gp58, colored from the N-terminus (blue) to the C-terminus (red), is shown fit to the unmodeled density. In the three panels, the top panel shows the C-terminal domain fit to the C1/C2 density, the middle panel shows the N-terminal domain fit to the D density, and the lower panel shows the C-terminal domain fit to the D density. (F) The model shows gp39 and gp58 based on the AlphaFold2 model and density fitting. See also Figure S2.

As mentioned, Syn5 has two regions of unmodeled density on the capsid surface just above gp39; at the periphery of gp39 and close to the two-fold axes, the unassigned density was referred to as the “C1/C2” density, and the unmodeled density at the center of the gp39 hexon (local six-fold axis) was referred to as “D” density (Figure 5A). In the original paper, the D density was assigned to gp55, while the C1/C2 density was assigned gp58. Both densities appear to have similar volumes; likewise, gp55 (156aa) and gp58 (168aa) have similar sequence lengths. As such, the original assignment of protein identity was based on the correlation of predicted versus observed secondary structure elements.

As already demonstrated with gp10 in ε15, predictive modeling has the ability to potentially identify and assign protein structure in a near-atomic resolution density map. Models for both gp55 (156aa) and gp58 (169aa) were constructed with AlphaFold2 and compared with the density (Figures 5B and 5C, Table 4). The overall quality of models varied considerably; the average pLDDT score of the five AlphaFold2 gp55 models was ∼75, whereas the gp58 models’ average pLDDT scores were closer to 45. Both the gp55 and gp58 models appeared to have two distinct domains. In gp55, the N-terminal domain consisted of a three-stranded β-sheets and a helix with a small C-terminal domain containing a short loop and a helix. gp58 on the other hand contained two 4 + turn helices and a long loop in the N-terminal domain that was connected to a C-terminal domain via a long loop that consisted of a five-stranded β-sheet. Interestingly, the different AlphaFold2 models for gp55 and gp58 varied primarily in the orientation of the two domains relative to each other, possibly suggesting a flexible conformation (Figure S2). None of the models fit their respective density, though the C-terminal domain of the gp58 AlphaFold2 models fit best to the unmodeled density near the two-fold axes (C1/C2 density), which was originally assigned to gp55 (Figures 5D and 5E). Moreover, fitting of the N-terminal domain of gp58, independently of the C-terminal domain, did show a good fit to the unmodeled density at the six-fold axes. No single AlphaFold2 model for gp58 reflected a conformation that allowed the occupancy of both the C1/C2 and D density sites, but fitting of the two domains and rebuilding of the connecting loop demonstrated a potential configuration consistent with the density map (Figure 5F). Not surprisingly, the modified AlphaFold2 gp58 model dramatically improved the average AlphaFold2 model fit to density.

### Mud Crab Reovirus

**Figure 6:**
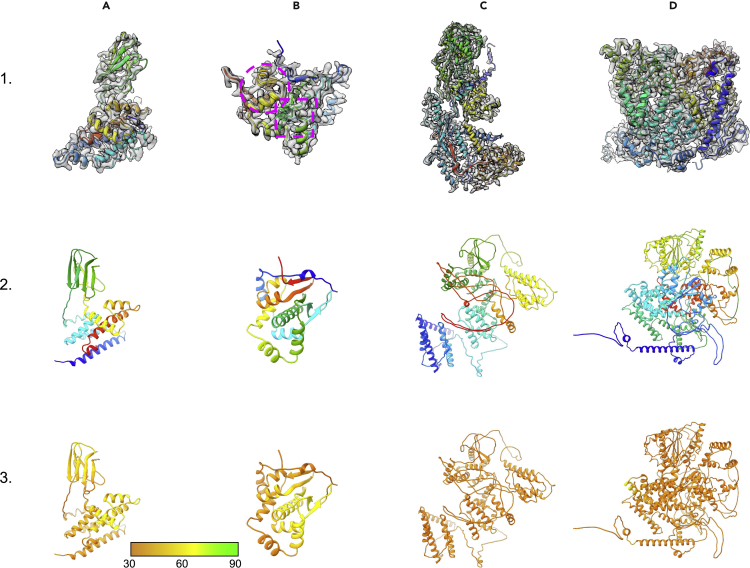
Modeling of MCRV (A–D) The experimentally determined and AlphaFold models for the structural proteins in the MCRV asymmetric unit are shown. Column (A) shows VP12, column (B) shows VP11, column (C) shows VP3, and column (D) shows the RdRP. In Row 1, the experimentally derived structures are shown colored from the N-terminus (blue) to the C-terminus (red) and superimposed on their corresponding cryoEM density. In Row 2, the corresponding AlphaFold2 models are shown colored from the N-terminus (blue) to the C-terminus (red). In Row 3, the corresponding AlphaFold2 models are shown colored based on pLDDT.

**Figure 7:**
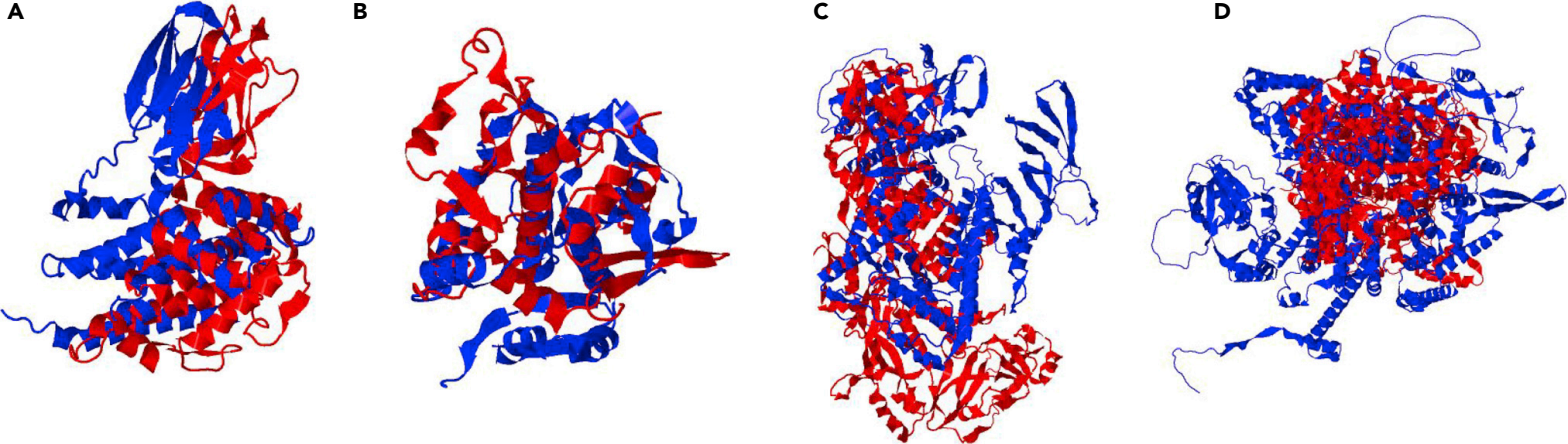
Structural Alignments of MCRV proteins (A–D) The AlphaFold2 models for the four MCRV structural proteins, (A) VP12 monomer, (B) VP11, (C) VP3, and (D) RdRP, are shown in blue superimposed on the corresponding reference structure in red. The structural alignment is derived from the TM-score alignment.

Members of *Reoviridae*, which infect a wide range of mammalian hosts, are characterized by a segmented double-stranded RNA (dsRNA) genome that is encapsulated by a proteinaceous shell. Single particle cryoEM and X-ray crystallography have been used to determine the structures of several *Reoviridae* family members with 9–11 segmented dsRNA genomes (Cheng et al., 2011; Lu et al., 1998; Nakagawa et al., 2003; Pan et al., 2021; Reinisch et al., 2000; Zhang et al., 2005, 2010). The structure of Mud Crab Reovirus (MCRV); Deng et al., 2012; Huang et al., 2012), with a 12-segment dsRNA genome, was determined to an ∼3.1-Å resolution using single particle cryoEM (PDB: 7XR2, EMDB: EMD-33403). The structure revealed MCRV to be organized as double layer capsid, composed of an RNA-dependent RNA polymerase (RdRP), surrounded by an inner capsid layer comprised of a pseudo-VP3 dimer, which, in turn, is surrounded by an outer capsid layer consisting of trimeric VP12 and a clamp-like protein, VP11 (Figure S3). Whereas there are structural homologues for VP3 and VP12, the structures of VP11 and, to a lesser extent, the RdRP are relatively unique. Each of the individual MCRV proteins were modeled with AlphaFold2 individually except VP12 that was modeled both as a monomer and a trimer (Table 5, Figures 6 and 7).

**Table 5:** MCRV model statistics

	VP3	VP11	VP12	VP12 trimer	RdRP
Composition					
Atoms	6,818	1,668	2,085	6,225	11,079
Residues	854	203	274	822	1,395
Molprobity score	3.67	3.77	2.97	3.50	3.72
Clash score	28.55	42.78	25.26	41.60	29.43
Ramachandran plot					
Outliers	20.77	14.43	12.13	10.17	21.18
Allowed	12.79	10.95	9.19	9.80	11.92
Favored	66.43	74.63	78.68	80.02	66.91
Rotamer outliers	13.58	13.68	2.68	7.59	15.05
Cbeta deviations	0.00	0.00	0.00	0.00	0.00
Bonds					
Length	0.026	0.025	0.024	0.023	0.026
Angles	4.838	3.989	3.421	3.508	4.673
pLDDT (mean)	30.31	36.86	43.99	40.09	29.12
Map–model CC	0.05	0.04	0.17	0.05	0.05
FSC (0.143)	6.9	3.2	3.9	7.5	12.7
TM-score	0.1283	0.2429	0.3076	0.2576	0.1455
RMSD (paired residues)	43.015	18.353	24.038	20.656	54.477

### MCRV VP12

The structure of VP12 is typical of *Reoviridae* outer capsid proteins in which the N- and C-terminal domains form a large helical domain close to the inner capsid surface; the middle portion of the VP12 sequence forms a β-sandwich and sits atop the α-helical domain (Figure 6A). Three subunits of VP12 are tightly bundled, forming a trimer in the viral capsid. Modeling of a single VP12 with AlphaFold2 (274aa) returned structures with a similar architecture – a β-sandwich domain on top of a mostly helical lower domain composed of the N and C termini. However, the overall organization and orientations of the domains are substantially different, resulting in RMSDs greater than 20 Å (Figure 7A). None of these AlphaFold2 models fit the VP12 cryoEM density map completely, with the best model having a map–model correlation value of 0.17 and an RMSD of 24.038 Å. Breaking the AlphaFold2 models into pieces, residues 1–104 (N-terminal helical domain), 105–192 (β-sandwich), and 193–274 (C-terminal helical domain), and comparing these domains with the experimentally determined model revealed where AlphaFold2 failed. The β-sandwich domain had an RMSD of 5.911 Å, whereas the C-terminal helical domain had an RMSD of 6.761 Å. In these domains, the overall fold was generally maintained with small deviations primarily localized to the loops. The major source of difference was sequence register shifts. Unlike these two domains, the N-terminal helical domain was substantially worse, with an RMSD of 19.141 Å. The overall architecture of the N-terminal helical domain was completely different from the experimentally determined model, resulting in models with substantial differences in the arrangement of the three domains with respect to each other.

The mean pLDDT scores for the AlphaFold2 VP12 models ranged from 39.78 to 43.82, putting the overall model quality into the low, unreliable category. However, further inspection of the β-sandwich domain and C-terminal helical domain revealed that the pLDDT scores for these domains were primarily in the range of 60– to 70, indicating increased reliability over the other portions of the structure. Taken together with the resulting models, it may be possible to identify, extract, and independently fit these domains to the density map. As noted, there are significant differences in these two domains among the various AlphaFold2 models and the experimentally derived structures and caution should be used if attempting to fit by parts.

### MCRV VP11

The second of the two outer capsid proteins in the MCRV capsid is VP11 (203aa). In contrast to VP12 (Figure 6B), VP11 is monomeric and does not have apparent sequence or structural analogue within *Reoviridae*. The structure of VP11 is predominantly α-helical with two small β-sheets. Prediction with AlphaFold2 returns models with relatively similar secondary structure though no predicted models resemble the experimentally determined model or fit the VP11 density. RMSDs for the top five AlphaFold2 models were 22.148, 17.583, 26.77, 18.097, and 21.937 Å. However, like VP12, portions of the predicted model did show similarity to the experimentally determined VP11 structure (Figure 7B). In particular, two AlphaFold2 models for residues 148–185 of VP11 (Figure 6B, dashed circle) had RMSDs of 2.970 and 2.754 Å; the other three AlphaFold2 models had RMSDs over 4 Å. For the two AlphaFold2 models that shared structural similarity to the experimental VP11 model, the three-helix motif was almost perfectly maintained with the largest differences owing to a small shift of the first helix relative to the other two helices. Interestingly, these two models also shared considerable structural homology to the experimentally derived structure of VP11 from residues 82–115 (Figure 6B, dashed rectangle). This domain contains a long, central alpha helix followed by a loop with a short β-strand running parallel to the long helical axis. Over these residues, the aforementioned two AlphaFold2 models had RMSDs of 4.189 and 5.053 Å, but captured the overall VP11 fold over this super-structure. Despite these similarities, these two models had overall RMSDs of ∼18 Å, largely owing to the fact that these structurally “conserved” regions were not orientated to each other in the same way as in the experimentally determined structure. As such, fitting and refining these two AlphaFold2 models to the density map based on either one of these conserved sub-domains resulted in a poor fit to density for the remaining portions of the models.

As with the previous examples, the overall model quality for the AlphaFold2 predictions were reasonable for VP11, but the pLDDT for all five AlphaFold2 models were well below 60, suggesting an overall low confidence in the models. Interestingly, one of the two AlphaFold2 models with sub-structure similarity did have an average pLDDT score near 70 for amino acids 55–120. However, the other model with structural similarity had similar pLDDT scores to models without the structural similarity over the same region.

### MCRV VP3A and VP3B

The inner capsid layer of MCRV is composed of a pseudo-VP3 dimer (VP3A and VP3B, 854 amino acids each) arranged on a T = 1 lattice (Figure 6C). The experimental models and density map clearly show that the majority of the VP3A and VP3B are the same except for the ∼80 N-terminal residues that adopt several unique conformations. The remaining structure of VP3 shares a common architecture seen other *Reoviridae* inner capsid proteins; a thin flat structure, roughly divided into an α-helical apical domain, a middle helical domain (carapace domain), and a dimerization domain composed of mainly β-strands. As with the other MCRV proteins, AlphaFold2 produced five predicted models for the VP3 sequence but none shared any significant structural homology to the experimentally derived structures for VP3A or VP3B; RMSDs ranged from 43.015 to 62.450 Å, with the best fit to density model having an RMSD of 43.015 Å (Figure 7C). Moreover, little structural conservation was seen for any domain or sub-structure in the AlphaFold2 models. The only similarity between the experimental and predicted VP3 models was the predominance of alpha helices. The pLDDT scores for all five AlphaFold2 models ranged from ∼25 to 40, except for the first ∼50 residues that have an average pLDDT score approaching 60. Interestingly, this region of VP3 in the experimental maps and models is highly variable and changes with the local environment.

### MCRV VP1

The last of the MCRV capsid proteins is VP1 or the RdRP (1,332 aa). The RdRP is located beneath, but slightly offset, of the five-fold axis and contacts three of the five VP3 pseudo-dimers. The N-terminal portions of at least four different VP3s interact with the RdRP in various manners. The RdRP is a common component in *Reoviridae* with members sharing moderate structural homology among the three different RdRP domains (N-terminal, polymerase, and C-terminal domains) (Ding et al., 2018; Pan et al., 2021; Tao et al., 2002; Zhang et al., 2003). However, the overall arrangement and structure of these domains in *Reoviridae* does vary. Again, the five AlphaFold2 models of the RdRP share almost no overall structural similarity with the experimentally derived map and model (Figures 6D and 7D). In fact, only one of the five models appears to be approximately the same dimensions as the RdRP density map. RMSDs for the five AlphaFold2 models ranged from 45.045 to 52.526 Å. None of the three RdRP domains shared any structural homology with the N-terminal, polymerase, and C-terminal domains, having RMSDs between 34.308 and 47.755, 33.020–50.376, and 31.848–51.817 Å, respectively. The pLDDT scores also support this observation, with the overall average pLDDT scores ranging from 20 to 40.

The relative failure of AlphaFold2 on all of the MCRV structural proteins can be clearly seen in the summary of model statistics in Table 5. All of the models have high clash scores and relatively high Molprobity scores, in addition to low confidence pLDDT scores and poor fit to density scores, suggesting that these models do not faithfully represent the actual experimentally derived models. Unlike the Syn5 examples where a low confidence model could be further refined and improved using the density map, the MCRV models are simply not accurate enough for further refinement.

## Discussion

Without a doubt, cryoEM and predictive modeling represent two rapidly emerging tools that will become mainstays in the toolkit for deriving the structure and function of macromolecules and complexes. The use of computational and predictive modeling in cryoEM may not necessarily be new ideas, but, until recently, the tools for generating robust and accurate models of proteins in complexes have been relatively limited and unreliable. As described here, the current generation of predictive modeling tools, like AlphaFold2, present an enormous leap in unlocking and rapidly advancing our understanding of macromolecular complexes, particularly when paired with experimental data. However tantalizing, the limitations of such an integrated approach have not been explored. The work described here helps to build that foundation and establish expectations of an integrated cryoEM-predictive modeling pipeline for model macromolecular structures at near atomic resolutions.

### Assessing AlphaFold2 with CryoEM community standards

From the analysis of the initial cryoEM challenge data sets, it would appear that, in large part, AlphaFold2 is very capable of producing reliable and robust models that can be used directly with cryoEM density maps in the construction of models for macromolecular assemblies. Of the 13 unique data sets, only one EMDB: EMD-22898 produced a result that did not immediately agree well with the density map, and, even then, only rigid-body, domain-level adjustments would have been necessary to properly fit the model into the map. In examining the entire set of maps and models as a whole, there were several trends that were noticeable (Table 1). As expected, there was a clear trend with RMSD and pLDDT; as pLDDT decreased, the model accuracy as assessed by RMSD with the reference structure decreased. As such, that decreased model accuracy resulted in poorer fits to density; likewise, higher pLDDTs generally were associated with better model fits to the density map. Another somewhat expected trend was that higher pLDDT scores were generally associated with better Molprobity scores. Interestingly, the Molprobity score obtained directly from the predictive models also had a correlation with a fit to density; better Molprobity scores generally meant a better fit to density. In the absence of reference structures, the Molprobity score and, to a lesser extent, clash score and Ramachandran outliers provide another metric for predicting how well the map and predictive model may agree.

The trends seen in the challenge data sets somewhat carry over to the three additional virus data sets: ε15, Syn5, and MCRV. In particular, the low pLDDT scores combined with the low Molprobity scores would likely have provided an indicator that the predicted MCRV models were of low confidence and likely not to agree well with the density. When compared with the experimentally derived structures, the high RMSD and low TM-scores for the predicted models further substantiates the failings on AlphaFold2 on this data set. However, a bit more interesting is ε15 gp10. All four of the models predicted for gp10 had reasonably good Molprobity and pLDDT scores, indicating that all of the sequences produced highly confident models. Despite having some of the lowest Molprobity and pLDDT scores of the four candidate sequences, the 130 amino acid sequence had the best fit to density. In Syn5, the gp39 model had a somewhat high Molprobity score as well as a borderline pLDDT score, yet the overall fold of the predicted model agreed well with the reference structure and the density map. As such, it is difficult to pinpoint a single metric for assessing the accuracy and reliability of predictive modeling in relation to cryoEM density modeling. Rather, the observed trends may simply be another tool in assessing predictive modeling.

Whereas the challenge data sets have played an important role in establishing the potential utility of a combined AlphaFold2 and cryoEM density modeling approach, as shown in the three additional virus cases, the expectations and potential limitations must still be addressed to fully realize the potential of integrating these complementary approaches.

### The good

In ε15, AlphaFold2 produced models of similar or better quality for the major capsid protein, gp7, than the then-state-of-the-art methods used to model the density map. It should be noted that, from a modeling standpoint, now and in the foreseeable future, cryoEM modeling tools will be able to produce better models directly from the density map than what was previously reported. However, the fact that a completely automated method for generating a structure directly from the sequence in the absence of any experimental data can produce a model so close to the experimentally derived map and model, in a matter of minutes, is a fantastic step forward.

Whereas the results for gp7 are encouraging, showing that AlphaFold2 could correctly predict the structure, perhaps more exciting are the results for gp10. As discussed, the original assignment of gp10 in the capsid was based on the limited biochemical evidence and secondary structure prediction of the possible candidate proteins. When predicting the structure of all possible 10–13KDa structural proteins in ε15, the original assignment of the gp10 density as the 111 amino acid protein is likely to have been incorrect. Of the four unique proteins ranging from 111 to 130 amino acids, only the 130 amino acid protein had a predicted structure consistent with the gp10 density. Furthermore, none of the other three structures appear to have any significant structural similarity to the experimentally derived fold for gp10. It is important to note that despite not having the correct sequence assignment the cryoEM derived model does have the same fold as the predicted structure for the 130 amino acid protein. As such, AlphaFold2 and other predictive methods could prove to be powerful for identifying and modeling unassigned density, as well as a new tool to probe and validate structure assignment in a cryoEM density map.

### The (not so) bad

Like ε15, Syn5 is a dsDNA bacteriophage and shares considerable structural similarity. As with the gp7, AlphaFold2 predictions of the major capsid protein of Syn5, gp39, are consistent with the experimentally derived structure. What is especially interesting in this example is the model quality and AlphaFold2’s own assessment of the prediction. With ε15 gp7, the average pLDDT score was 77.80 with some regions going into the low 90s. Using AlphaFold2’s own guidelines, models with pLDDT scores above 70 are considered “good.” Syn5 gp39 had an average pLDDT score of 60.99, making the model a borderline low confidence prediction. In fact, large portions of the structure have pLDDT scores below 60, yet the overall fold of the model agrees well with the density and requires only the same “tweaking” as the ε15 gp7 model to maximize fit to density.

Continuing with the similarities to ε15, the capsid of Syn5 contains at least one additional structural protein. Unlike ε15 though, the extra capsid protein density was never modeled and only assigned as gp55 for the density near the two-fold axis (C1/C2) and gp58 for the density at the six-fold axis (D) based on secondary structure prediction. Neither of the densities is sufficient to account for the 151 or 169 amino acids in gp55 and gp58, respectively. However, the C-terminal portion of the AlphaFold2 model for gp58 is consistent with the density at the two-fold, whereas the N-terminus contains two helices that are approximately the same size as the density at the six-fold axes. It is interesting to note that all five AlphaFold2 models for gp58 are similar, varying primarily on the relative orientation of the N-terminal domain in relation to the C-terminal domain. When fit separately, the two domains of the AlphaFold2 gp58 model fit the density well, while still maintaining a similar overall distance between the domains, suggesting that the extra density in the Syn5 capsid may in fact be gp58. However, despite the improved fit to density, the resolution of the density map and the lack of any corroborating biochemistry makes it impossible to determine the validity of this assignment. Further conflating the analysis, the average pLDDT score for gp58 is below 50, suggesting the AlphaFold2 model is of very low quality. On the other hand, the average pLDDT score for gp55 is considerably better at almost 77, though none of the AlphaFold2 models for gp55 fit the unmodeled density. In this regard, modeling with AlphaFold2 cannot provide any robust annotation or modeling of the density, though it can help to provide a testable hypothesis for determining the composition of the unmodeled density.

Whereas the model and predictive metrics for the ε15 examples closely resembled what we saw in the challenge data sets, the Syn5 examples represented an example similar to EMDB: EMD-22898. Here, the pLDDT scores were relatively low, but the models were mostly correct and could be refined to fit the density, even if it meant the model had to be broken and refined as domains.

### The ugly

For both Syn5 and ε15, AlphaFold2 was able to provide some meaningful modeling of the capsid proteins. However, in the case of MCRV, none of the four capsid proteins modeled with AlphaFold2 resulted in a structure that was consistent with the density map or experimentally determined model. At best, only small stretches of residues shared any structural similarity to the experimentally determined models. Furthermore, all MCRV models had average pLDDT scores below 50, again indicating that these models were generated with very low confidence. In terms of modeling, this example represents one of the worst possible cases – predictive modeling provides no reasonable structure or starting point for further model building/refinement. Moreover, the map–model correlation is so bad that one would expect that even naive model building would steer away from using these structures as model building templates.

What is particularly interesting about the MCRV case is that the structure of several *Reoviridae* capsids and capsid proteins have been determined and deposited in the Protein DataBank. And, whereas sequence similarity among the corresponding capsid proteins of *Reoviridae* is relatively low, structural similarities persist throughout *Reoviridae*. At the time of these experiments, the MCRV structures were not published and the models were not in any structural database, and therefore would not have been included in any of the training/template data for AlphaFold2. However, the existence of numerous related structures should have been sufficient to model the MCRV proteins with AlphaFold2.

### What’s next?

Obvious from these and other results, AlphaFold2 has shown enormous potential in contributing to our understanding of macromolecular structure and function. The examples shown here are of single proteins, but as we all know, proteins rarely work independently and are often parts of large dynamic complexes. The ability to solve large macromolecular complexes makes cryoEM an invaluable tool for structure determination. So where does AlphaFold2 and predictive modeling stand in terms of modeling macromolecular complexes? Whereas not originally designed for complexes, newer versions of AlphaFold2 have now begun to emerge targeting complexes (Evans et al., 2021). Here, we tested a version of AlphaFold2_Advanced that expands upon the DeepMind AlphaFold2 notebook. AlphaFold2_Advance does allow for modeling of small complexes, a feature we used in modeling EMDB: EMD-22898, as well as ε15 and MCRV (see below). Our ultimate goal is to not just model individual proteins, but understand their relationship to each other and potential interactions that drive function in a complex.

To assess AlphaFold2_Advanced’s current ability to derive accurate models of complexes, we attempted to model a gp7–gp10 complex in ε15. In the ε15 capsid, there is essentially a 1:1 correspondence of gp10 to gp7, and the position of the gp7–gp10 interaction is conserved throughout the hexon on the outermost surface of the capsid, just above the large three stranded β-sheet. Here, two gp10s from two adjacent asymmetric units appear to form a head-to-tail dimer; this configuration led to gp10 being referred to as a “staple.” For the purposes of this work, we attempted to model a single gp7 and a single gp10 as a complex. AlphaFold2_Advanced returned five potential configurations of the gp7–gp10 complex, and for the most part, the models for the individual subunits were nearly identical to those obtained when modeling gp7 and gp10 alone with AlphaFold2 (Figure S4). Of the five models, only the third ranked model showed a similar orientation to the published structure and density map. In this complex, gp7 was within 1-Å RMSD of the true gp7 location in the map, whereas gp10 was shifted ∼8 Å away from gp7 in addition to a slight lateral rotation (∼15 degrees). The individual AlphaFold2 models for gp7 and gp10 were fit to the density map and had an average map value of 0.314 and a map–model correlation of 0.478. Illustrating the shift in fit, the AlphaFold2_Advanced predicted gp7–gp10 complex had an average map value of just 0.2067 and a map model correlation of 0.3465.

In MCRV, VP12 forms a trimer, making up the majority of the outer capsid surface. We attempted to run AlphaFold2_Advanced to predict the structure of a homotrimeric VP12 trimer. As noted in the modeling of the individual VP12 subunits, AlphaFold2 was not able to produce models consistent with the density map and experimentally derived models. This was in large part owing to poor modeling of the N-terminal domain and the repercussions it had on the β-sandwich and C-terminal domains. In an attempt to constrain the orientation of these domains relative to each other, we attempted to model three VP12 subunits, the naturally occurring oligomeric state in the intact capsid, with AlphaFold2 (Figure S5). As with the case of the monomer, the AlphaFold2_Advanced models for a trimeric VP12 did not resemble the experimentally derived model or the density map. Moreover, the trimer models did not improve the orientation of the three VP12 domains.

Whereas the modeling of individual proteins was a success, AlphaFold2_Advanced still largely failed to model assemblies of proteins. The largest limiting factors in the prediction are likely size/scale of the prediction and what constitutes the basic structural unit that accommodates all possible interactions. Predictive models for the gp10 and gp7 in ε15 produced structures with dramatically different subunit orientations with respect to each other. Only one of the models recapitulated the approximate location of the two subunits, though the overall model statistics for the five different orientations were not indicative of which was the “best” model. Expanding the predictive models to include multiple copies of gp7 and gp10 were beyond the size of the current AlphaFold2_Advanced limit. Likewise, proper modeling of VP12 may require the addition of multiple VP12, VP11, and VP3 subunits to produce models consistent with the structures generated directly from the experimental density map. However, such complexes are far too large for current implementations of AlphaFold2_Advanced.

Regardless of AlphaFold2_Advanced’s ability to correctly predict the structure of complexes at this time, the results are encouraging and hint at the potential of future predictive modeling tools. Ultimately, whereas certain trends were noticed in regards to predictive modeling accuracy, experimental data are paramount in obtaining and validating the “correct” structure. Certainly, future predictive modeling tools could greatly benefit from the incorporation of biochemical and structural information to help constrain “model space” and validate alternate conformations when modeling protein complexes.

## Conclusion

Based on the examples shown, predictive modeling with AlphaFold2 has reached a level whereby it can produce individual protein structures of sufficient quality that they can be directly used in the interpretation and modeling of large complexes from near-atomic resolution density maps. In general, the pLDDT score is a good guide to the overall model quality and accuracy; clash scores and Ramachandran outliers also provide a limited estimation of model accuracy. However, there are a number of examples discussed here where model confidence scores are not sufficient indicators of potential model accuracy. In Syn5, model confidence scores were relatively poor, yet the gp39 and gp58 models fit the observed density relatively well after limited refinement. Conversely, in ε15, the four candidate protein sequences for the unmodeled capsid density were all highly confident models based on the pLDDT scores. The only way to determine the “correct” structure was to fit these models, each corresponding to a unique sequence, to the density map and assess the fit.

So, to the question, “Is there any reliable metric for predicting model accuracy?” the answer is rather straightforward. pLDDT and model quality metrics are reasonable metrics for judging the quality of the model, but the accuracy of predictive models must still be evaluated based on the agreement with experimental data. With an ever-growing library of known structures, predictive modeling may eventually achieve the needed level of accuracy to model dynamic protein structures and complexes. Until then, AlphaFold2 and other predictive modeling, despite all their successes, cannot replace experimental methods nor are there any transparent mechanisms to determine when/why/how a predictive model might have failed. Nevertheless, predictive modeling can still be an incredibly powerful tool to generate testable hypotheses and guide future experimental structure determination studies.

### Limitations of the study

The work described here utilizes a publicly available version of AlphaFold2_advanced and all models described in this work were generated using the current version of the software with default options unless otherwise noted. Updates to or new versions of AlphaFold2_advanced, local installations of AlphaFold2 or non-default options may result in similar but slightly different models and statistics compared with the ones in this study. However, unless a significant alteration of the algorithm or major change to the training set is implemented, the overall results should be qualitatively similar.

## STAR★Methods

### Key resource table

**Table:** 

REAGENTS OR RESOURCE	SOURCE	IDENTIFIER
**Deposited data**		

EMDataResource	https://www.emdataresource.org/	EMD-2847, EMD-5623, EMD-5776, EMD-7770, EMD-22898, EMD-2842,EMD-6422, EMD-0406, EMD-20027, EMD-6000, EMD-30210, EMD-2677, EMD-5954,EMD-33403, EMD-5678
EM Validation Challenges	https://challenges.emdataresource.org/	

**Software and algorithms**		

AlphaFold2_Advanced	Version 2022/2/4 (Jumper et al., 2021)	https://colab.research.google.com/github/sokrypton/ColabFold/blob/main/beta/AlphaFold2_advanced.ipynb
ChimeraX	Version 1.3.8 (Pettersen et al., 2021)	https://www.cgl.ucsf.edu/chimerax/
Phenix	Version 1.19.2 (Liebschner et al., 2019)	https://phenix-online.org/
Coot	Version 0.9.6 (Emsley et al., 2010)	https://www2.mrc-lmb.cam.ac.uk/personal/pemsley/coot/

### Resource availability

#### Lead contact

Further information and requests for either resources or reagents should be directed to and will be fulfilled by the lead contact Matthew L Baker (Matthew.L.Baker@uth.tmc.edu).

#### Materials availability

Requests for materials generated in this study should be directed to and will be fulfilled by the lead contact Matthew L Baker (Matthew.L.Baker@uth.tmc.edu).

### Method details

In assessing predictive modeling methods for constructing atomic models in cryoEM density maps, we selected a set of community established standard density maps from the past three cryoEM modeling challenges: the 2021 Ligand Model Challenge, the 2019 Model Metrics Challenge and the 2016 Model Challenge (https://challenges.emdataresource.org/). These three challenges yielded 12 unique biological datasets, representing a variety of macromolecular organizations, with resolutions from 1.8 to 4.5Å. For each of the datasets, the sequences corresponding to all chains were extracted and used for predictive modeling except for EMD: EMD-2847, a 70s ribosome structure with over 50 individual protein subunits. For EMD: EMD-2847, six random chains were selected, bringing the total to 24 unique protein chains.

For the purpose of this work, we generated all predictive models using the AlphaFold2_advanced Google Colab notebook, available at https://colab.research.google.com/github/sokrypton/ColabFold/blob/main/beta/AlphaFold2_advanced.ipynb). Unless otherwise stated, each run of AlphaFold2 used only a single sequence (less than 1400 amino acids) and only included modeling of a single individual subunit. Multiple sequence alignments were generated with mmseqs2(Mirdita et al., 2019). All other parameters were set to the default settings, except for in the case of VP11 of MCRV. For VP11, the number of recycles was increased to 48 (the max number) from 3. Increased numbers of recycles in the other MCRV data sets resulted in errors related to running out of RAM. Five models were generated for each target sequence with the pLDDT (predicted Local Distance Difference Test) score (Mariani et al., 2013), a per-residue confidence score between 0 and 100, maped to the B-factor column of the resulting PDB files. Generally, pLDDT scores above 90 are considered “very confident,” pLDDT scores between 70 and 90 are considered “confident,” pLDDT scores between 50 and 70 are “low confidence” and scores below 50 are considered “very low confidence” and may be unstructured in isolation.

All models were initially rigid body fit to the corresponding density map using UCSF’s Chimera (Pettersen et al., 2004). While the five resulting AlphaFold2 models were examined, the best fitting model to the corresponding density was selected for further refinement. Refinement of the model to the map was performed using Phenix’s real-space refinement tool (Afonine et al., 2018; Liebschner et al., 2019); resolution was set to the stated resolution of the map and the “run” parameter included local_grid_search, minimization_global, morphing, adp and simmulated_annealing. After refinement, the maps and models were visualized in Coot (Emsley et al., 2010); regions with obvious clashes, bad fit to density or modeling errors were corrected and subjected to another round of Phenix real-space refinement without morphing or simulated annealing. While run times varied based on the size/sequence of the model, the entire modeling process typically took ∼1–1.5 h on an Apple M1 Mac Mini to generate a model directly from the sequence and refine it against the density map.

### Quantification and statistical analysis

Maps and models were visualized in UCSF’s Chimera and ChimeraX (Pettersen et al., 2021). Assessment and model comparison was performed using Chimera (model vs. model RMSD), Phenix (map/model statistics) and MolProbity (model evaluation) (Chen et al., 2010; Williams et al., 2018).

## Data Availability

• All density maps shown in this work are available through the EMDataResource. Selected maps and models in the cryoEM modeling challenges are also made available directly from the EM Validation website. • This paper does not report original code. • Any additional information required to reanalyze the data reported in this paper is available from the lead contact upon request.
