# Supplementary material for: AlphaFold2 and CryoEM: Revisiting CryoEM modeling in near-atomic resolution density maps
Source: iScience. 2022 May 30;25(7):104496. doi: 10.1016/j.isci.2022.104496 (PMC9207676; doi:10.1016/j.isci.2022.104496)
Supplement: Document S1. Figures S1–S5 [file mmc1.pdf]

iScience, Volume *25*

Supplemental information

AlphaFold2 and CryoEM: Revisiting CryoEM modeling in near-atomic resolution density maps

Corey F. Hryc and Matthew L. Baker

A.

Sequence length (candidate)	Sequence
111	MKTVNMKTGTDSFVGEDGKPETKDQYPWGLRITLDNESLQRLGLNAKSLPAV GDSVSVMAMANVCSVSTRTTDHGEDNYVELQITDIGLAPQKRDDAKELKDAFY PDGEDD
112	MAKSPAERKAAQRARQYAAGERKLELVLDGQELEMLARNCTARRHGRAPYD MSEYIALLIRQDDARVRGRIKSISANRCGKCGDSLPVKSCPCDGDSACWVTRG WHETKLSV
121	MSDKDIESEIQAKGLTAPRVTPQRIESIIASEHYFTAQDGVIGSQYCHIPNGEISV GPHSLDLLTFCVMTLANGFTVTGESACASPENFDAEIGRKIARENAVNKIWMLE GYLLKQKLSEQ
130	MLLDQQALFSAAQAITATAVSTNVIDTGSSKDVGKYGDIPLLIQVVEAFNTLTSLT VTVQTDDNSSFSSATDVISMVIPLASLTVGYKTPVITLPMKLERYIRLNYTVTGTA PTTGKVTAGIVGGVQTNV

B.

Sequence length (candidate)	111	112	121	130
111		5.5%	7.6%	14.6%
112			22.3%	15.6%
121				10.9%

Figure S1. GP10 candidate sequences. Related to Figure 3. Four ~12kDa sequences were identified as potential candidate proteins for the gp10 density. The sequences are shown in (A). (B) Pairwise alignments among the four sequences show little sequence identity.

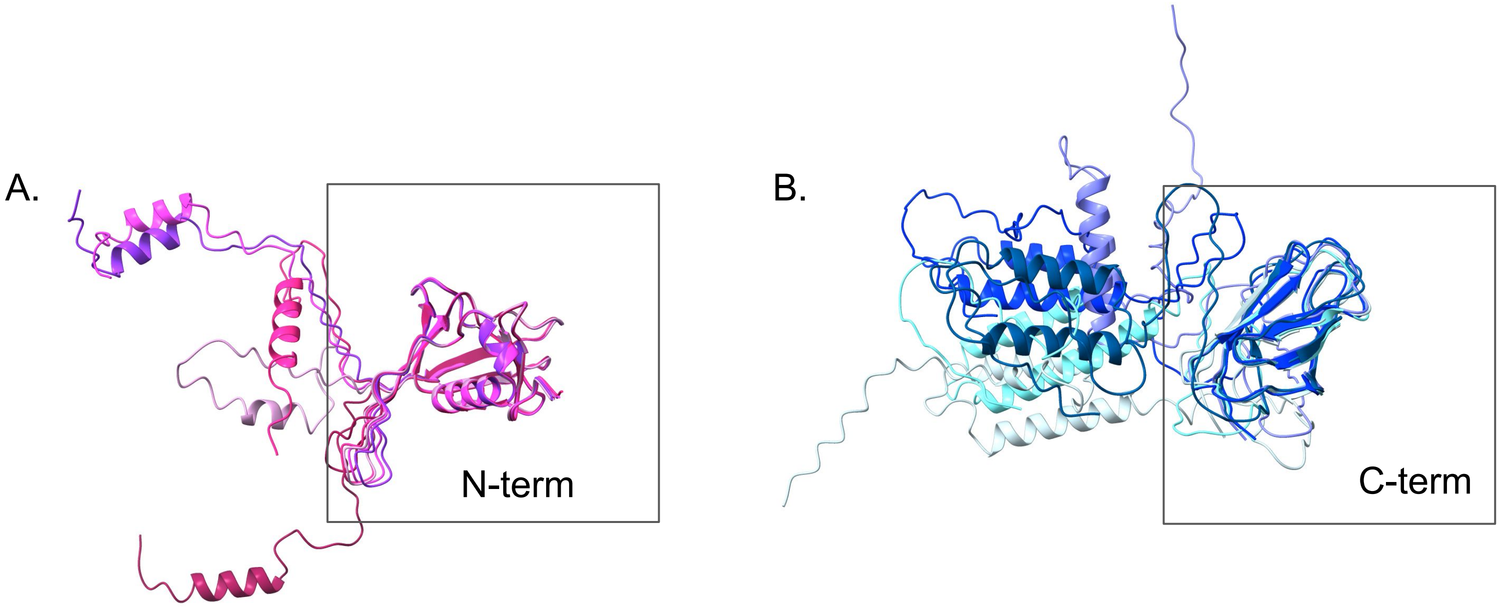

Figure S2. AlphaFold2 model of gp55 and gp58 in Syn5. Related to Figure 5. Five AlphaFold2 models were generated for gp55 (A) and gp58 (B). Models for gp55 were aligned to each other; each model is colored in a shape of pink or purple in (A). Models for gp58 were aligned to each other; each model is colored in a shape of pink or purple in (B).

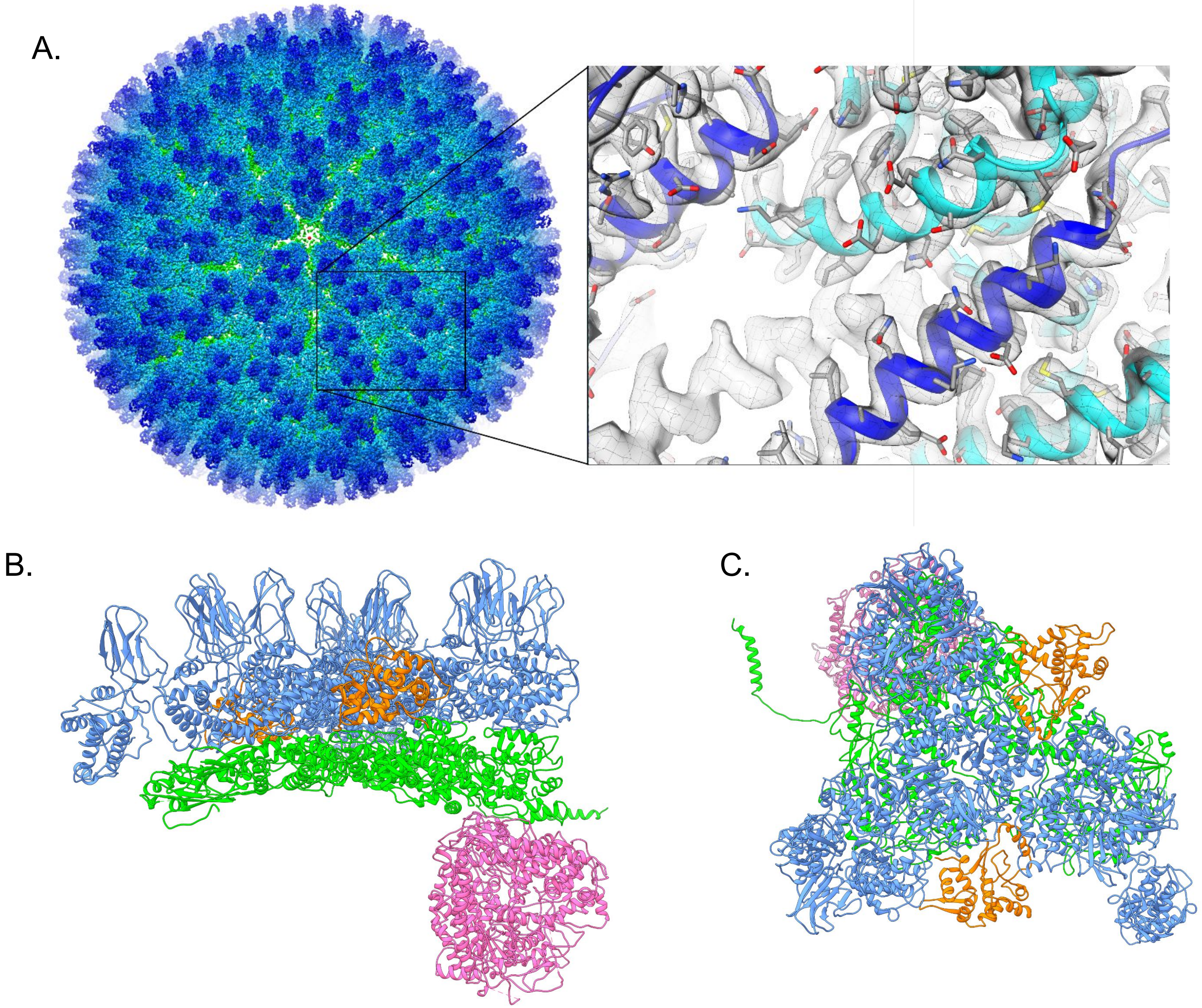

Figure S3. Density map and model of MCRV. Related to Figure 6. (A) The MCRV cryoEM density map is shown colored radially. A zoomed in view of the capsid reveals high-resolution features and the corresponding model. An asymmetric unit from MCRV is shown in a side view (B) and a top view (C). In this figure, VP11 is shown in orange, VP12 is shown in purple, VP3 is shown in green and the RdRP is shown in bright pink.

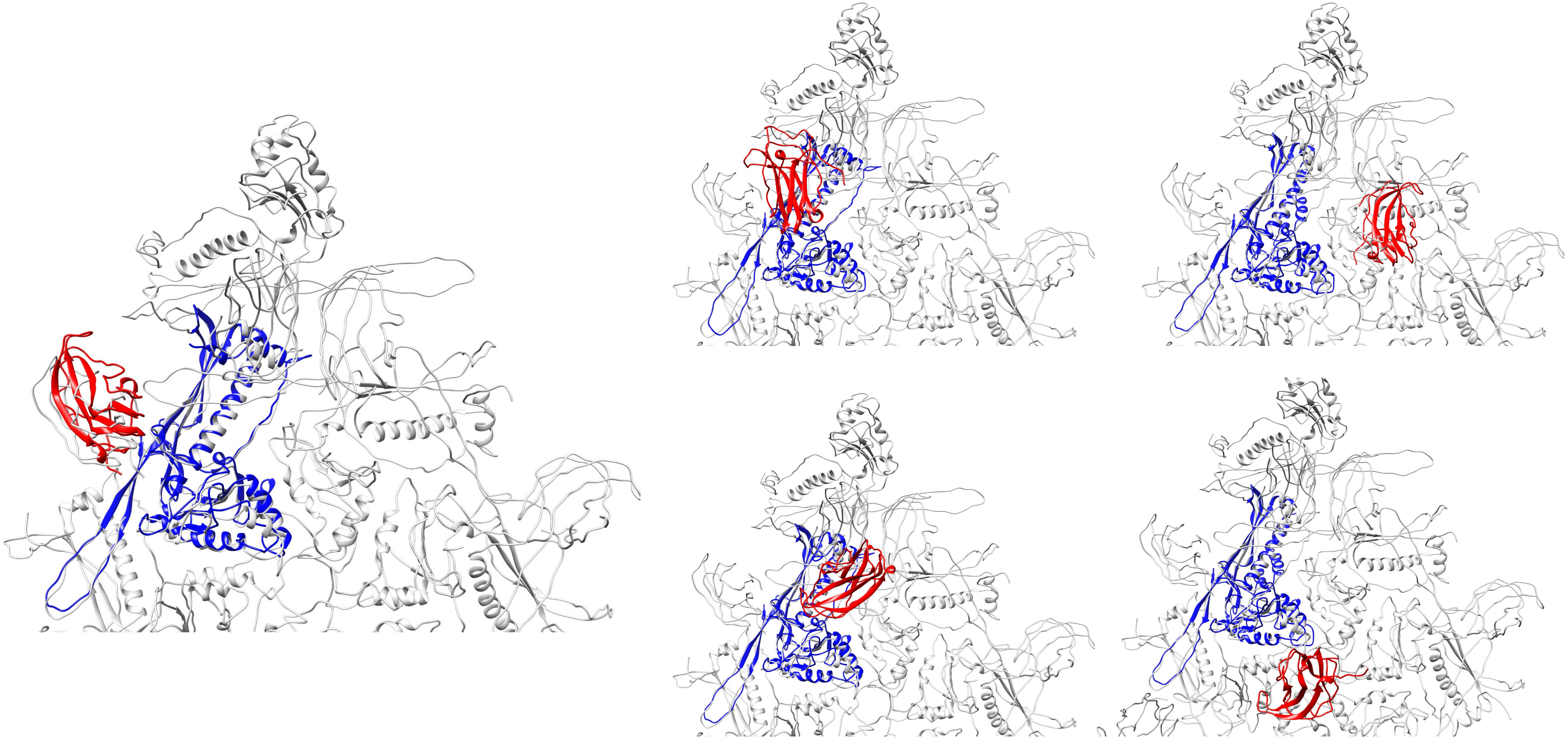

Figure S4. AlphaFold2 model of a gp7/gp10 complex. Related to Figure 2 and 3. Five AlphaFold2 models for a gp7/gp10 complex are shown with gp7 in blue and gp10 in red. The experimentally derived models for gp7 and gp10 in a hexon are shown in grey. Of the five AlphaFold2 models, only one (left) positioned gp7 and gp10 close to the experimentally determined positions.

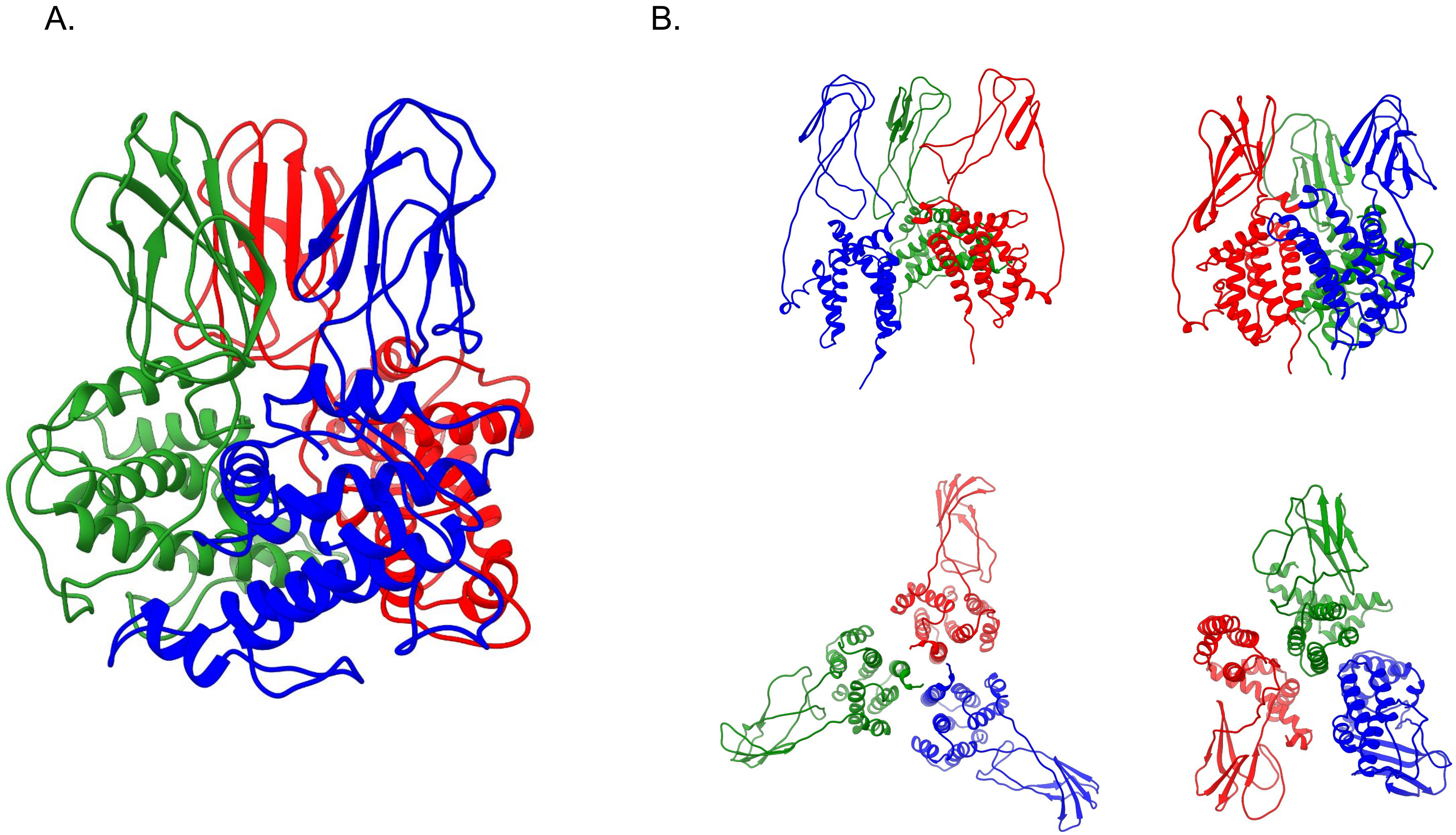

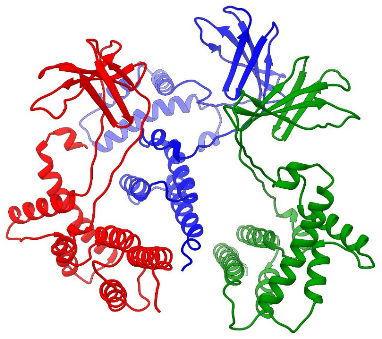

Figure S5. AlphaFold2 models for a VP12 complex. Related to Figure 6. (A) A VP12 timer model derived from the density map is shown colored by chain. (B) The five AlphaFold2 models for a VP12 trimer are shown colored by chain.
